# Phase I dose-ascending, safety, and pharmacokinetics study of APC148, a novel metallo-β-lactamase inhibitor, in healthy volunteers

**DOI:** 10.1128/aac.00426-26

**Published:** 2026-06-17

**Authors:** Bjørg Bolstad, Ragnar Hovland, Johan Bylund, Erik Rein-Hedin, Sandra Kuusk, Bjørn Klem, Pål Rongved

**Affiliations:** 1 AdjuTec Pharma AS, Oslo, Norway; 2 Clinical Trial Consultants AB, Uppsala, Sweden; 3 Department of Surgical Sciences, Plastic Surgery, Uppsala University 174469, Uppsala, Sweden; Providence Portland Medical Center, Portland, Oregon, USA

**Keywords:** APC148, β-lactamase inhibitor, metallo β-lactamases, phase 1, pharmacokinetics

## Abstract

APC148 is a novel metallo-β-lactamase inhibitor with broad activity against Ambler class B enzymes, including NDM, VIM, and IMP. It is being developed for patients with serious infections caused by multidrug-resistant, gram-negative bacteria. APC148 is combined with the broad-spectrum β-lactam antibiotic meropenem and the serine-β-lactamase inhibitor avibactam, which targets Ambler class A, C, and some class D (OXA-48-like) enzymes. In combination with meropenem and avibactam, APC148 demonstrated superior *in vitro* activity against a global, multidrug-resistant collection of Enterobacterales, showing its promising activity against β-lactamase-producing pathogens. In this randomized, placebo-controlled, first-in-human study, the safety, tolerability, and pharmacokinetics of APC148 were evaluated in healthy adults. Single doses ranging from 50 mg to 760 mg APC148 were administered intravenously over 3 h to 46 participants across six dose groups. APC148 was well-tolerated at all dose levels. All adverse events were of mild intensity, and no serious adverse events or adverse events leading to study or treatment discontinuation occurred. The pharmacokinetics of APC148 were dose-proportional, with low plasma clearance, low-to-moderate volume of distribution, and a mean plasma half-life of 2.6 h. APC148 is well-tolerated and represents a promising candidate in the fight against antibiotic-resistant bacteria.

**CLINICAL TRIALS:**

This study is registered with ClinicalTrials.gov as NCT06360640.

## INTRODUCTION

Infections caused by antimicrobial-resistant pathogens represent a significant and growing global disease burden. Between 2025 and 2050, 39.1 million deaths attributable to antimicrobial resistance (AMR) and 169 million deaths associated with AMR are forecasted (1). Carbapenem-resistant gram-negative Enterobacterales, including *Klebsiella pneumoniae* and *Escherichia coli,* are ranked among the highest-priority bacterial pathogens by the World Health Organization (2).

Broad-spectrum β-lactam antibiotics such as carbapenems (e.g., meropenem) and cephalosporins (e.g., cefepime) are hydrolyzed by serine- and metallo-β-lactamases (SBLs and MBLs) produced by multidrug-resistant bacteria (3, 4). While combinations of β-lactam and SBL inhibitors are on the market, no approved therapies combined β-lactams with specific MBL inhibitors (5, 6). APC148 is a novel selective zinc-inhibitor targeting Ambler class B MBLs (e.g., NDM, VIM, and IMP) that restores β-lactam antibiotic activity without having intrinsic antibacterial effects (7). As both SBLs and MBLs can be expressed in the same pathogen, simultaneous inhibition of both enzymes is essential (8–10).

In an *in vitro* susceptibility testing of a global collection of 176 MBL- and SBL-producing Enterobacterales isolates (SENTRY, 2019–2022), APC148 was tested in combination with either cefepime-avibactam or meropenem-avibactam and compared to pipeline and approved antibiotics. APC148 showed superior activity against all other products with a MIC_90_ of 0.12 µg/mL (11, 12). This high activity of meropenem-avibactam with APC148 against multidrug-resistant pathogens was recently confirmed in an Indian collection of 303 isolates of *E. coli* and *K. pneumoniae* (11, Internal report, 2026). These *in vitro* results have been confirmed by *in vivo* studies and support the development of APC148 for the treatment of patients with serious infections caused by multidrug-resistant gram-negative pathogens.

Based on preclinical data, a first-in-human study was conducted to evaluate the safety, tolerability, and pharmacokinetics of single-ascending doses of APC148 in a randomized, placebo-controlled study in healthy adults.

## RESULTS

### Disposition and demographics

A total of 46 participants were randomized into six escalating dose cohorts. Cohort 1 included six participants, four randomized to APC148 and two to placebo. Cohorts 2 through 6 each included eight participants, six randomized to APC148 and two to placebo. All participants received a single 3 h intravenous infusion of APC148 or placebo and completed the study according to the protocol. Demographic characteristics are summarized in Table 1. Baseline characteristics were generally comparable across the cohorts. Overall, a higher proportion of men than women was enrolled in the study.

**TABLE 1:** Demographics

Parameter	Placebo (*n* = 12)	APC148 dose					
		50 mg (*n* = 4)	150 mg (*n* = 6)	300 mg (*n* = 6)	450 mg (*n* = 6)	650 mg (*n* = 6)	760 mg^*a*^ (*n* = 6)
Sex (*n* [%])							
Female	4 (33)	2 (50)	2 (33)	0	1 (17)	2 (33)	1 (17)
Male	8 (67)	2 (50)	4 (67)	6 (100)	5 (83)	4 (67)	5 (83)
Age (yrs) (mean [SD])	39.1 (14.4)	52.3 (9.6)	50.7 (9.4)	31.0 (9.1)	39.0 (15.1)	40.0 (15.3)	34.7 (13.6)
Race (*n* [%])							
White	12 (100)	4 (100)	4 (67)	6 (100)	6 (100)	6 (100)	5 (83)
Asian			1 (17)				1 (17)
Multiple			1 (17)				
Weight (kg) *(*mean [SD])	75.0 (9.1)	75.3 (4.7)	81.1 (20.1)	82.0 (9.6)	77.4 (9.4)	75.9 (10.6)	73.0 (7.1)
BMI (kg/m^2^) (mean [SD])	23.9 (3.0)	24.7 (3.0)	26.2 (4.1)	24.6 (3.3)	25.3 (3.3)	24.5 (2.4)	23.5 (3.3)
eGFR^*b*^ (mL/min/1.73m^2^) (median [min, max])	89.5 (68, ≥90)	≥90 (84, ≥90)	≥90 (77, ≥90)	≥90 (80, ≥90)	88 (79, ≥90)	86 (81, ≥90)	≥90 (82, ≥90)

^*a*^ Following dose group 5 (650 mg), where one participant approached the estimated maximum exposure of ACP148, the increment to the next dose group was reduced to 760 mg.

^*b*^ eGFR, estimated glomerular filtration rate based on serum creatinine, calculated using the creatinine-based revised Lund Malmø GFR-estimating equation. The laboratory reports eGFR values only up to 90 mL/min/1.73 m^2^; values of 90 are presented as ≥90.

### Safety and tolerability

All participants tolerated APC148 well. Twenty-three of the 46 participants, 20/34 (59%) on APC148, and 3/12 (25%) on placebo reported 33 adverse events in total. There were no deaths, serious adverse events, or adverse events leading to study or treatment discontinuation. All adverse events were mild in intensity, and most (28/33 events) were assessed as unlikely related to treatment with APC148. There were no clinically relevant mean changes from baseline in safety laboratory parameters, vital signs or physical examinations between APC148 and placebo, and no apparent trends indicating a dose-dependent relationship with respect to these safety parameters. Except for one instance of erythema, all local tolerability adverse events were assessed as being associated with the study procedures, and not APC148. In general, 12-lead electrocardiogram (ECG) assessments did not reveal any safety concerns. A mild, transient increase in the heart rate corrected QT interval (QTcF) was indicated in the highest dose groups (450, 650, and 760 mg); there was no clear dose-response relationship, and there was a large inter-individual variation within and between dose groups.

A total of five adverse events, all of mild intensity, were assessed as related to APC148, including erythema, rash, muscular weakness, headache, and a transient and asymptomatic ECG QT prolongation. The QT prolongation, assessed using QTcF, was observed in one female participant in the 760 mg dose cohort. (Table 2). The maximum QTcF value was 469 ms, observed 3 h and 30 min after the start of the infusion. On the day prior to infusion, QTcF was 446 ms, and at pre-dose, it was 433 ms.

**TABLE 2:** Subjects with at least one treatment-related adverse event

Adverse event	No. (%) for each group							
	Placebo (***n*** = 12)	APC148 dose						
		50 mg (*n* = 4)	150 mg (*n* = 6)	300 mg (*n* = 6)	450 mg (*n* = 6)	650 mg (*n* = 6)	760 mg (*n* = 6)	Pooled (*n* = 34)
Subjects with at least one adverse event					2 (33)	1 (17)	2 (33)	5 (15)
Erythema					1 (17)			1 (2.9)
Rash					1 (17)			1 (2.9)
ECG QT prolonged							1 (17)	1 (2.9)
Muscular weakness						1 (17)		1 (2.9)
Headache							1 (17)	1 (2.9)

### Pharmacokinetics

**Fig 1:**
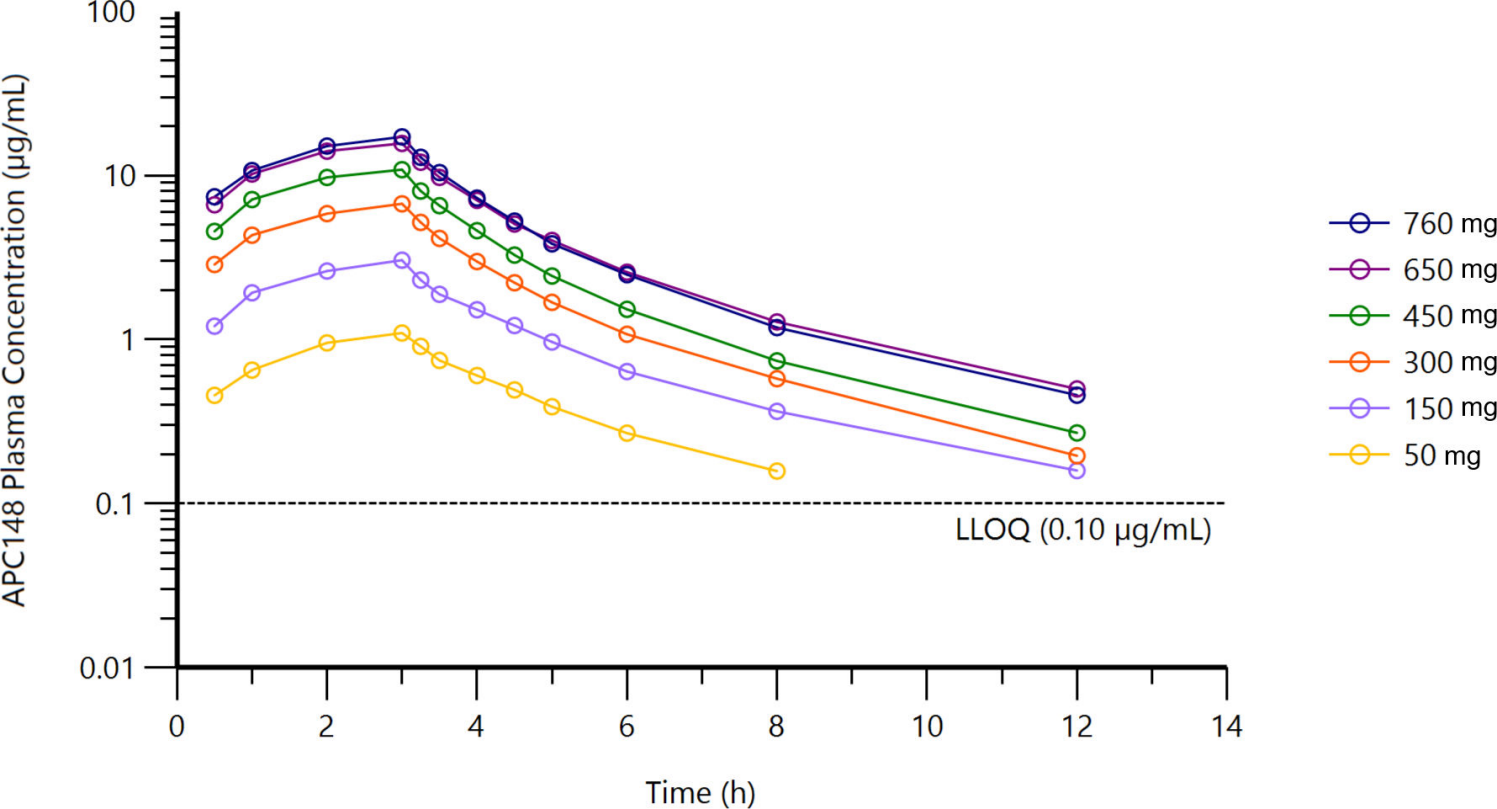
Geometric mean APC148 plasma concentration for cohorts 1–6, versus time following a single dose. Logarithmic concentration scale. LLOQ, lower level of detection.

APC148 demonstrated a consistent pharmacokinetic profile across the 50–760 mg dose range. The estimated slopes for both C_max_ and AUC_inf_ were close to 1 (1.044 for C_max_ and 0.9829 for AUC_inf_), indicating dose proportionality over the investigated dose range (50–760 mg). The mean half-life ranged from 2.3 to 3.0 h (Table 3). Peak plasma concentrations were observed at the end of the 3-h infusion for all dose levels (Fig. 1). Mean plasma clearance (CL) was low and dose-independent, and the mean volume of distribution at steady state (Vss) was in the low-to-moderate range.

**TABLE 3:** Pharmacokinetic parameters for APC148^*a*^

Cohort	Dose (mg)	N	C_max_ (µg/mL)	AUC_inf_ (h*µg/mL)	CL (L/h)	V_ss_ (L)	T_1/2_ (h)	F_e_ (%)	CL_r_ (L/h)^*b*^
1	50	4	1.10 (13.3)	4.78 (11.3)	10.5 (11.3)	27.5 (16.5)	2.3 (12.9)	38.2 (11.1)	4.0 (21.6)
2	150	6	3.04 (19.9)	12.9 (20.7)	11.6 (20.7)	32.9 (21.8)	3.0 (12.5)	45.4 (6.1)	5.3 (17.2)
3	300	6	6.72 (15.6)	25.7 (13.7)	11.7 (13.7)	25.4 (16.0)	2.5 (13.2)	33.4 (39.4)	3.9 (52.8)
4	450	6	10.9 (13.8)	40.3 (14.5)	11.2 (14.5)	22.1 (21.0)	2.4 (14.3)	63.8 (25.4)	7.1 (37.9)
5	650	6	15.7 (11.7)	61.4 (16.0)	10.6 (16.0)	24.8 (21.2)	2.8 (36.9)	67.9 (16.8)	7.2 (24.4)
6	760	6	17.2 (9.8)	63.5 (10.5)	12.0 (10.5)	25.4 (16.1)	2.7 (28.7)	59.8 (6.9)	7.2 (13.7)

^*a*^ Statistics show geometric means (percent geometric coefficient of variation). C_max_, maximum plasma concentration; AUC_inf_, area under the plasma concentration versus time curve from time zero extrapolated to infinity; CL, total body clearance; V_ss_, volume of distribution at steady state; T_1/2_, terminal elimination half-life; F_e_, fraction of drug excreted unchanged in urine; CL_r_, renal clearance.

^*b*^ Different bioanalytical methods were used for cohorts 1–3 and 4–6.

APC148 concentrations were measured in urine fractions collected over 0–24 h. The fraction of the administered APC148 dose excreted unchanged in urine (Fe%) ranged from 33% to 68% over the same interval. The mean renal clearance (CLr) values ranged from 4 L/h to 7 L/h (Table 3). Two different bioanalytical methods were used for urine sample analysis. Samples from cohorts 1–3 (50–300 mg) were analyzed using the validated method, whereas samples from cohorts 4–6 (450–760 mg) were analyzed using an exploratory method after it was observed that the original validated assay had an insufficient quantification range for higher urine concentrations of APC148.

## DISCUSSION

APC148 is a selective zinc inhibitor that inhibits MBLs and has no intrinsic antibacterial activity (7). APC148 restores the *in vitro* and *in vivo* activity of meropenem against multidrug-resistant MBL-producing Enterobacterales (7). When meropenem is combined with APC148 and the SBL inhibitor avibactam, the combination shows potent activity against both MBL- and SBL-producing pathogens, as shown in *in vitro* and *in vivo* studies (11, 12).

As a first step in the clinical development of APC148, a single dose-escalating study was conducted in healthy volunteers to assess safety, tolerability, and pharmacokinetics. Fewer participants were included in the lowest active treatment dose cohort because no clinically relevant pharmacological effect was expected at this dose level. Cohorts receiving 150–760 mg in the active treatment arm were expanded to six participants, as this was considered appropriate to better characterize safety and pharmacokinetic variability. Pharmacokinetic analysis indicated an average half-life of 2.6 h, supporting the use of combinations with β-lactam antibiotics such as carbapenems and cephalosporins. The slightly higher renal clearance values observed in cohorts 4–6 compared to cohorts 1–3 are more likely due to differences in bioanalytical methods than to a true dose-related change in renal clearance. This interpretation is supported by the similar total plasma clearance observed across cohorts. Overall, the results indicate that renal excretion is the primary route of elimination. This finding is consistent with the animal studies and suggests possible utility in the treatment of complicated urinary tract infections. The highest administered dose was generally well-tolerated, with one female in this group presenting a transient, asymptomatic ECG QT prolongation. The present clinical trial was not designed or powered to formally assess QTcF effects with statistical significance. Therefore, the ECG findings should be interpreted with caution. No consistent trend in QTcF was observed. Based on the available data, it cannot be determined whether the observed changes were reflected to APC148 or reflected individual outliers, regression to the mean, or inherent physiological variability, or other non-treatment-related factors. Further characterization of QTcF effects will be conducted in future dedicated studies.

The potent inhibition of MBLs observed in preclinical studies, combined with the pharmacokinetics and safety profile, supports further development of APC148. Combinations of β-lactams antibiotics and β-lactams inhibitors, including APC148, address a rapidly growing unmet medical need for the treatment of patients with MBL-producing infections.

## MATERIALS AND METHODS

### Study design

This was a first-in-human, randomized, double-blind, placebo-controlled, single-ascending dose (SAD) trial of APC148 in healthy adults. The study enrolled participants at a single center in Sweden between September 2024 and March 2025

A single 3 h intravenous infusion of APC148 was administered to all participants in six sequential cohorts. The administered doses were 50, 150, 300, 450, 650, and 760 mg. The initial 50 mg safety cohort included four active treatment participants and was expanded to six active treatment participants in subsequent cohorts with greater pharmacological and clinical relevance. Following dose group 5, where one participant approached the estimated maximum exposure of APC148, the increment of the subsequent dose escalation was reduced.

The first two participants in each cohort were dosed in a sentinel fashion, that is, one received APC148, and one received placebo as randomized. The safety and pharmacokinetic data for a completed dose cohort were reviewed by a safety review committee before making the decision to escalate to the next dose cohort.

### Participants

Healthy males and females of non-child-bearing potential, aged ≥18 to ≤60 years, with a body mass index ≥18.5 and ≤30.0 kg/m^2^ and adequate renal function were considered eligible for trial participation. Adequate renal function was defined as serum creatinine within normal limits (i.e., ≤upper limit of normal), and a creatinine estimated glomerular filtration rate (eGFR) ≥80 mL/min/1.73 m^2^ for participants aged 18–50 years, and eGFR ≥60 mL/min/1.73 m^2^ for participants ≥51 years. eGFR was calculated using the revised Lund-Malmö GFR-estimating equation. A participant could only be enrolled within one dose cohort.

### Safety assessments

Safety was assessed based on the occurrence of adverse events, local tolerability, evaluation of vital signs, 12-lead electrocardiograms (ECG), laboratory parameters (chemistry, hematology, coagulation, and urinalysis), and physical examination that was performed throughout the study. Safety was followed for 7 days post-dose.

### Pharmacokinetic assessments

Blood samples for the determination of APC148 in plasma were collected pre-dose and at 30 min and at 1, 2, 3, 3.25, 3.5, 4, 4.5, 5, 6, 8, 12, 24, and 48 h after the start of drug administration. For all cohorts, urine was collected pre-dose and at intervals of 0–6, 6–12, and 12–24 h after the start of the administration.

### Bioanalytical methods

Human plasma with K2EDTA (dipotassium ethylenediaminetetraacetic acid) was processed using protein precipitation and assayed using the UPLC-MS/MS system. The calibration ranges of 100–150,000 ng/mL were used. Urine samples were also analyzed using the UPLC-MS/MS method. The bioanalytical method used for the urine analysis of samples from cohorts 1–3 (50–300 mg) was fully validated over the concentration range of 100–150,000 ng/mL. Due to several urine samples of cohort 4 (450 mg) exceeding the upper limit of quantification, a new exploratory method was developed in the concentration range of 1,000 to 2,000,000 ng/mL and applied for samples collected in cohorts 4–6 (450–760 mg).

### Pharmacokinetic analysis methods

Individual participant plasma and urine PK parameters were evaluated using non-compartmental analysis methods (Phoenix WinNonlin, Certara, USA). Calculated plasma PK parameters were, among others, C_max_, AUC_0–inf_, CL, V_ss_, and T_1/2_. Non-compartmental analysis was based on the actual sampling times recorded during the trial. The urine PK parameters were Ae (amount of unchanged drug excreted in urine during the 0–24 h interval), Fe (fraction of IV administered drug that is excreted into urine), and CL_R_ (renal clearance). Dose proportionality based on C_max_ and AUC_inf_ was determined using a power model based on log-transformed (natural logarithm) pharmacokinetic parameters.
